# Association between rehabilitation after reperfusion treatment and in-hospital mortality: Results from a national registry study

**DOI:** 10.3389/fneur.2022.949669

**Published:** 2022-09-14

**Authors:** Shengde Li, Shiyuan Fang, Dingding Zhang, Yixiu Lu, Longde Wang, Bin Peng

**Affiliations:** ^1^Department of Neurology, Peking Union Medical College Hospital, Peking Union Medical College, Chinese Academy of Medical Sciences, Beijing, China; ^2^Laboratory of Complex Severe and Rare Diseases, Peking Union Medical College Hospital, Peking Union Medical College, Chinese Academy of Medical Sciences, Beijing, China; ^3^The General Office of Stroke Prevention Project Committee, National Health Commission of the People's Republic of China, Beijing, China

**Keywords:** stroke rehabilitation, thrombolysis, endovascular therapy, mortality, bigdata

## Abstract

**Background:**

There is no effective regimen to reduce the mortality of patients treated with intravenous thrombolysis or endovascular therapy (EVT). Therefore, we aimed to examine whether sequential therapy by rehabilitation could effectively reduce the in-hospital mortality of patients treated with reperfusion therapy.

**Methods:**

This prospective registry study included patients with ischemic stroke who were treated by intravenous thrombolysis or endovascular therapy at Stroke Center Work Plan in China between 1 October 2018 and 31 July 2020. The patients were divided into 2 groups: those with (IRT+) or without (IRT–) inpatient rehabilitation therapy (IRT). The primary outcome was all-cause in-hospital mortality. We used Cox proportional hazards models and conducted a propensity score matching analysis to calculate hazard ratios (HRs) for mortality in the thrombolysis-only and EVT groups.

**Results:**

Of the 189,519 patients in the thrombolysis-only group, 35.7% were women, and the median (interquartile range, IQR) age, onset-to-needle time, and follow-up time were 66 (57–74) years, 165 (119–220) min, and 9 (5–12) days, respectively. Among the 45,211 patients in the EVT group, 35.9% were women, and the median (interquartile range, IQR) age, onset-to-puncture time, and follow-up time were 66 (56–74) years, 297 (205–420) min, and 11 (6–16) days, respectively. In the thrombolysis-only group with a median (IQR) initial National Institutes of Health Stroke Scale (NIHSS) score of 6 (3–11), 105,244 patients (55.5%) treated with IRT had significantly lower all-cause in-hospital mortality [0.6 vs. 2.3%; adjusted HR 0.18 (95% confidence interval (CI) 0.16–0.2)] than those without IRT. In the EVT group with a median (IQR) initial NIHSS score of 15 (10–20), 31,098 patients (68.8%) treated with IRT also had significantly lower all-cause in-hospital mortality [2 vs. 12.1%; adjusted HR, 0.13 (95% CI 0.12–0.15)]. IRT remained significantly associated with reduced in-hospital mortality in sensitivity, subgroup, and propensity score matching analyses among both the thrombolysis-only and EVT groups.

**Conclusion:**

Among the patients with ischemic stroke treated with intravenous thrombolysis or endovascular therapy, sequential therapy by rehabilitation was associated with lower all-cause in-hospital mortality. These findings suggest the necessity of promoting inpatient rehabilitation therapy after reperfusion in patients with ischemic stroke.

## Introduction

Acute ischemic stroke (AIS) causes millions of deaths and disability in China and other countries every year (1, 2). Intravenous thrombolysis and endovascular therapy (EVT) have been proven effective and recommended to improve 3-month functional outcomes after AIS (3). However, the proportions of poor outcome and death remain high after reperfusion therapy. Two recent large scale meta-analyses reported that 3-month mortality rates in patients treated with intravenous alteplase and EVT were 17.9 and 15.3%, respectively (4, 5). However, compared with the control group, both intravenous alteplase and EVT failed to reduce mortality risk (4, 5). Rehabilitation is an essential part of stroke care and has been associated with lower risk of mortality and disability (6, 7). However, the relationship between inpatient rehabilitation therapy (IRT) after reperfusion therapy and mortality in patients with AIS has not been well-delineated.

To improve the national quality of stroke care, China launched the Stroke Center Work Plan with evidence-based support (8, 9). A set of best practice strategies and auditing criteria was introduced to hospitals, and clinical data including rehabilitation and outcome were collected prospectively.

The aim of this study is to determine whether IRT after reperfusion therapy is associated with lower in-hospital mortality among patients with AIS. We conducted a national prospective observational cohort study to determine the effect of IRT on in-hospital mortality based on the Stroke Center Work Plan.

## Methods

The data supporting this study are available from the corresponding author upon reasonable request.

### Data source and study population

The Stroke Center Work Plan was launched by the China Stroke Prevention Project Committee in 2016 to provide evidence-based inpatient stroke care in 31 provinces in China aiming at improving clinical outcome of stroke. Details of the Stroke Center Work Plan were published elsewhere (8, 9). All clinical data are uploaded on the Bigdata Observatory Platform for stroke in China (BOSC, https://www.chinasdc.cn/) (Supplementary Figure S1) (10). All the data in our study were obtained from the database of Stroke Center Work Plan. Our study was approved by the Ethics Committee of Peking Union Medical College Hospital, with a waiver of informed consent (S-K988).

Trained hospital personnel were instructed to collect data of AIS patients receiving intravenous thrombolysis or EVT, covering demographics, stroke onset time, hospital arrival time, reperfusion therapy initiation time, stroke severity, rehabilitation status, in-hospital outcomes, etc. (8, 9). The diagnosis of AIS was made according to the International Classification of Diseases, Ninth Revision, Clinical Modification (ICD-9-CM). Hospital characteristics were also recorded. Each stroke center, project offices in each province, and the national project committee monitored the data in real time and verified the quantity and quality of the reports monthly. Hospitals that failed to pass 3 consecutive audits were disqualified from the reporting system.

**Figure 1:**
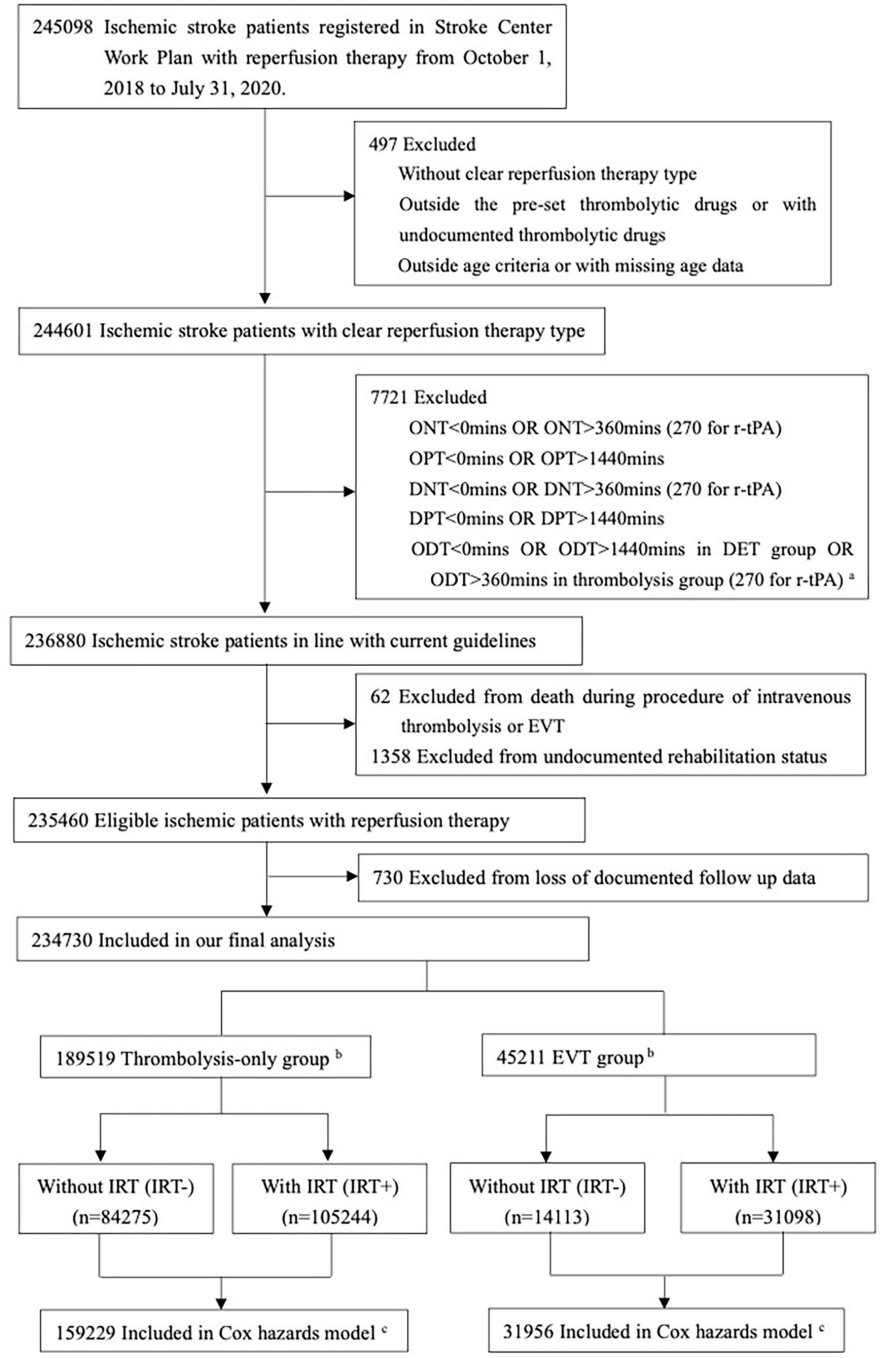
Flow of patients in our study. ^a^r-tPA: recombinant tissue plasminogen activator. ^b^Thrombolysis-only group for patients with intravenous thrombolysis and without endovascular therapy; EVT group for patients with endovascular therapy and with/without intravenous thrombolysis. ^c^Excluded from Cox proportional hazards models because of missing data on patient-level characteristics and follow-up times.

The study inclusion criteria required patients to (1) be aged between 14 and 99 years old, (2) have a diagnosis of AIS, (3) have received intravenous thrombolysis or EVT, (4) have a stroke onset time (if unavailable the time of arrival at hospital or admission and initiation time of intravenous thrombolysis or EVT as an alternative) from 1 October 2018 to 31 July 2020, and (5) have received recombinant tissue plasminogen activator (r-tPA), urokinase, anistreplase, reteplase, tenecteplase, or recombinant human pro-urokinase for those with intravenous thrombolysis. The exclusion criteria were (1) unknown reperfusion therapy type, (2) no documented rehabilitation data, (3) reperfusion time against Chinese guidelines, i.e., those with onset-to-needle time > 360 min (details are shown in Figure 1), (4) death during the procedure of intravenous thrombolysis or EVT, and (5) undocumented outcome at discharge.

### Procedure

AIS patients with reperfusion therapy were divided into 2 groups, those with and without IRT, i.e., IRT+ and IRT-, respectively. The IRT+ group was also defined as receiving sequential therapy of rehabilitation. This observational study recorded the real-world choice of IRT but did not explore its underlying cause as to why certain patients did not receive IRT. The index date (i.e., start of the follow-up) was defined as the stroke onset time. The follow-up time was defined as from the index date to the date of discharge or death.

IRT was categorized into five interventions in our study: (A) acupuncture or massage, (B) physical therapy (PT), (C) occupational therapy (OT), (D) speech therapy, and (E) other interventions of rehabilitation, including cognitive training, swallowing therapy, psychotherapy, or physiotherapy based on traditional Chinese medicine. PT and OT were recommended as post-stroke rehabilitation interventions in clinical practice, aiming at improving the function, mobility, and ability to carry out activities of daily living (7, 11–13). A previous study suggested that acupuncture might be beneficial in improving functional outcome (14); therefore, it was also included as an intervention of IRT in our study (7). The endpoint of a follow-up in our analysis was 31 August 2020.

To further clarify concerns for acupuncture, swallowing therapy, and other interventions (7, 14, 15), the interventions of IRT were divided into two subtypes: 1) type-A IRT (IRT-A): those with PT or OT; 2) and type-B IRT (IRT-B): those with rehabilitation (described above) but without PT or OT.

Both intravenous alteplase and EVT were recommended as effective interventions of reperfusion therapy. However, the characteristics, time window and etiologies were different among selected patients receiving intravenous alteplase or EVT. Moreover, EVT with or without intravenous alteplase was more effective in improving functional independence in AIS caused by occlusion of the proximal anterior circulation than only intravenous alteplase (5, 16). Thus, the eligible patients in our study were stratified into two groups to test the effect of IRT: (A) the thrombolysis-only group composed of patients with intravenous thrombolysis and without EVT, and (B) the EVT group composed of patients with EVT regardless of intravenous thrombolysis.

### Variables

The definitions of drugs of intravenous thrombolysis, ΔNIHSS 24 h, levels of change in NIHSS score at 24 h, reperfusion status after EVT (poor, good, or unknown), EVT types, periods of COVID-19 pandemic, and intracranial hemorrhage (ICH) are shown in Supplementary Table S1. Based on the quality of medical care and management, the hospitals in our study were graded according to three levels: class A, class B, and class C (details in Supplementary Table S1). Class A indicated the highest level. Rapid neurological improvement (RNI) was defined as ΔNIHSS 24 h ≥ 8, which was reported as an early indicator of a favorable outcome (17, 18).

### Outcomes

The pre-specified primary outcome was in-hospital death of all causes, which was recorded and verified in the information monitoring system. Time to death was measured starting from the index date.

### Statistical analyses

Categorical variables were presented as frequencies and percentages, while continuous variables with non-normal distributions were presented as median and interquartile range (IQR). Pearson χ2 test and Wilcoxon test were conducted for categorical and continuous variables between the IRT- and IRT+ groups, respectively.

A Kaplan-Meier curve with visual inspection was used to verify the assumption of proportional hazards in the Cox analysis. Then, Cox proportional hazards models were used to examine the association between IRT and in-hospital mortality in the thrombolysis-only and EVT groups. Confounders were evaluated by the change in estimate approach (19). Hazard ratios with 95% confidence interval (CI) are reported.

In addition, we assessed whether the association between IRT and mortality differed in certain subgroups by testing the IRT-by-subgroup interaction effect. Stratified Cox proportional hazards regressions were performed for subgroup analysis. We performed propensity score matching (PSM) to reduce the confounding effects of non-randomized IRT assignment. Demographic and clinical covariates were included in the PSM analyses. The nearest-neighbor algorithm with 1:1 matching and a caliper width of 0.02 was used. The balance of covariates was assessed by standardized differences. The MatchIt and Stddiff packages in R version 3.6.3 were used to perform the PSM. The HRs of IRT were also calculated.

All the statistical tests were two sided and had a significance level of *p* < 0.05. All the statistical analyses, except for the PSM, were conducted using SAS version 9.4 (SAS Institute Inc.).

## Results

### Patient characteristics

Of the 245,098 ischemic patients registered on the Stroke Center Work Plan database, 10,368 (4.2%) were excluded based on the enrollment criteria and 234,730 patients were included in our final analysis (Figure 1 and Supplementary Table S2), covering 2,220 hospitals. A total of 189,519 (80.7%) received only intravenous thrombolysis, and 45,211 (19.3%) were treated with EVT. Patients' demographic and clinical characteristics are shown in Table 1, with limited missing data (Supplementary Tables S3, S4). In the thrombolysis-only group, patients' median age was 66 years, 35.7% were women, and the median ONT was 165 min. In the EVT group, patients' median age was 66 years, 35.9% were women, and the median OPT was 297 min (Table 1 and Supplementary Table S5). The structures and contents of IRT in both thrombolysis-only and EVT groups are shown in Supplementary Table S6.

**Table 1:** Demographic and clinical characteristics of patients with thrombolysis only and endovascular therapy.

	**Thrombolysis-only group** ^a^				**Endovascular therapy group** ^a^			
	**Overall**	**Without IRT**	**With IRT**	***P*** **-value**	**Overall**	**Without IRT**	**With IRT**	***P*** **-value**
Patients	189,519	84,275 (44.5)	105,244 (55.5)		45,211	14,113 (31.2)	31,098 (68.8)	
Age, y	66 (57–74)	66 (56–73)	67 (57–74)	<0.0001	66 (56–74)	66 (57–74)	66 (56–74)	0.0002
Sex				0.0022				<0.0001
Male	121,799 (64.3)	54,479 (64.7)	67,320 (64.0)		28,965 (64.1)	8,854 (62.7)	20,111 (64.7)	
Female	67,715 (35.7)	29,793 (36.4)	37,922 (36.0)		16,245 (35.9)	5,259 (37.3)	10,986 (35.3)	
Hospital level				<0.0001				<0.0001
Class A	99,106 (52.3)	40,838 (48.5)	58,268 (55.4)		38,667 (85.5)	11,890 (84.3)	26,777 (86.1)	
Class B	26,506 (14.0)	12,101 (14.4)	14,405 (13.7)		4,095 (9.1)	1,275 (9.0)	2,820 (9.1)	
Class C	63,878 (33.7)	31,312 (37.2)	32,566 (30.9)		2,448 (5.4)	947 (6.7)	1,501 (4.8)	
TOAST				<0.0001				<0.0001
LAA	93,188 (49.2)	39,231 (46.6)	53,957 (51.3)		27,749 (61.4)	8,374 (59.4)	19,375 (62.3)	
CE	20,645 (10.9)	8,755 (10.4)	11,890 (11.3)		13,169 (29.1)	4,224 (30.0)	8,945 (28.8)	
SAO	65,984 (34.8)	30,970 (36.7)	35,014 (33.3)		1,994 (4.4)	569 (4.0)	1,425 (4.6)	
SOC	1,469 (0.8)	689 (0.8)	780 (0.7)		710 (1.6)	235 (1.7)	475 (1.5)	
SUC	8,200 (4.3)	4,615 (5.5)	3,585 (3.4)		1,569 (3.5)	699 (5.0)	870 (2.8)	
Reperfusion time, mins^b^	165 (119–220)	165 (119–220)	165 (119–221)	0.0478	297 (205–420)	285 (200–400)	300 (205–425)	<0.0001
EVT type				…				0.0596
BT	…	…	…		13,190 (29.2)	4,033 (28.6)	9,157 (29.5)	
Direct EVT	…	…	…		32,021 (70.8)	10,080 (71.4)	21,941 (70.5)	
Initial NIHSS score	6 (3–11)	5 (3–10)	6 (4–11)	<0.0001	15 (10–20)	16 (11–22)	15 (10–19)	<0.0001
Initial NIHSS score				<0.0001				<0.0001
0–4	74,999 (39.9)	38,688 (46.5)	36,311 (34.7)		3,033 (7.0)	1,023 (7.7)	2,010 (6.6)	
5–9	57,477 (30.6)	22,614 (27.2)	34,863 (33.3)		6,118 (14.1)	1,560 (11.8)	4,558 (15.1)	
10–14	29,408 (15.7)	10,631 (12.8)	18,777 (18.0)		11,331 (26.0)	2,889 (21.8)	8,442 (27.9)	
15–20	16,614 (8.9)	6,702 (8.1)	9,912 (9.5)		12,933 (29.7)	3,803 (28.8)	9,130 (30.2)	
≥21	9,231 (4.9)	4,572 (5.5)	4,659 (4.5)		10,089 (23.2)	3,954 (29.9)	6,135 (20.3)	
Initial mars	1 (0–3)	1 (0–3)	2 (0–4)	<0.0001	2 (0–4)	3 (0–4)	2 (0–4)	<0.0001
NIHSS score at 24 h	3 (1–7)	2 (0–4)	4 (2–8)	<0.0001	11 (5–18)	13 (4–24)	11 (6–16)	<0.0001
ΔNIHSS 24 h				<0.0001				<0.0001
Moderate–severe deterioration	6,773 (3.9)	2,378 (3.3)	4,395 (4.4)		3,989 (10.8)	1,854 (18.3)	2,135 (8.0)	
Mild deterioration	7,916 (4.6)	2,233 (3.1)	5,683 (5.7)		2,099 (5.7)	627 (6.2)	1,472 (5.5)	
Stable	40,275 (23.2)	16,743 (22.9)	23,532 (23.5)		9,063 (24.6)	2,650 (26.1)	6,413 (24.0)	
Mild improvement	102,067 (58.9)	44,420 (60.7)	57,647 (57.6)		14,458 (39.3)	2,890 (28.5)	11,568 (43.3)	
RNI	16,253 (9.4)	7,371 (10.1)	8,882 (8.9)		7,230 (19.6)	2,129 (21.0)	5,101 (19.1)	
Antiplatelet within 48 h				<0.0001				<0.0001
No	9,064 (4.8)	4,292 (5.1)	4,772 (4.5)		3,716 (8.2)	1,344 (9.5)	2,372 (7.6)	
Yes	149,822 (79.0)	63,109 (74.9)	86,713 (82.3)		29,864 (66.1)	7,929 (56.2)	21,935 (70.5)	
Unknown	30,633 (16.2)	16,874 (20.0)	13,759 (13.2)		11,631 (25.7)	4,840 (34.3)	6,791 (21.8)	
Statin				<0.0001				<0.0001
No	12,031 (6.4)	8,429 (10.4)	3,602 (3.6)		6,055 (14.3)	3,626 (27.9)	2,429 (8.3)	
Yes	170,576 (89.9)	72,841 (89.6)	97,735 (96.4)		36,276 (85.7)	9,378 (72.1)	26,898 (91.7)	
ICH				0.0003				<0.0001
No	183,161 (96.6)	81,307 (96.5)	101,854 (96.8)		41,293 (91.4)	12,312 (87.3)	28,981 (93.3)	
Yes	6,353 (3.4)	2,965 (3.5)	3,388 (3.2)		3,875 (8.6)	1,783 (12.7)	2,092 (6.7)	
GIH				<0.0001				0.0045
No	188,229 (99.3)	83,630 (99.2)	104,599 (99.4)		45,015 (99.7)	14,031(9.5)	30,984 (99.7)	
Yes	1,285 (0.7)	642 (0.8)	643 (0.6)		153 (0.3)	64 (0.5)	89 (0.3)	
Duration of follow up, d	9 (5–12)	7 (4–11)	10 (7–13)	<0.0001	11 (6–16)	6 (2–12)	12 (8–18)	<0.0001

BT, bridging thrombolysis; CE, cardio embolism; DET, direct endovascular therapy; GIH, gastrointestinal hemorrhage; ICH, intracranial hemorrhage; IRT, inpatient rehabilitation therapy; LAA, large artery atherosclerosis; mars, modified ranking score; NIHSS, National Institutes of Health Stroke Scale; RNI, rapid neurological improvement; SAO, small artery occlusion; SOC, stroke of other determined cause; SUC, stroke of undetermined cause.

a Categorical variables: frequencies and percentages; continuous variables with non-normal distributions: median and interquartile range (IQR).

b Onset-to-needle time (ONT) in patients with only intravenous thrombolysis; onset-to-puncture time (OPT) in patients with endovascular therapy.

In the thrombolysis-only group, patients in the IRT+ group had older age, more severe neurologic deficit, higher proportion of large artery atherosclerosis stroke, longer reperfusion time (ONT), and longer duration of hospitalization than those in the IRT-group. The percentage of receiving standard r-tPA, aspirin, and statins was markedly higher in the IRT+ group, while the IRT+ group showed slightly lower rates of intracranial hemorrhage and gastrointestinal hemorrhage.

In the EVT group, the baseline demographic and clinical data were heterogeneous between the IRT- and IRT+ groups. In brief, the reperfusion time (OPT) was longer and the proportion of large artery atherosclerosis stroke was higher in the IRT+ group. However, patients in the IRT+ group were slightly younger and had milder neurologic deficit, higher reperfusion rate (modified TICI score of 2b or 3, 60 vs. 49.4%), higher percentages of receiving aspirin and statins, and lower rates of intracranial hemorrhage and gastrointestinal hemorrhage. Besides, more patients were treated in class-A hospitals, and the proportion of moderate-severe deterioration was markedly lower in the IRT+ group.

After PSM, 113,864 patients were 1:1 matched in the IRT- and IRT+ groups in the thrombolysis-only group, and 15,420 patients in the EVT group were 1:1 matched in the IRT- and IRT+ groups (Supplementary Table S7). The distribution of the estimated propensity score of matched patients in the thrombolysis-only and EVT groups is shown in Supplementary Figures S2, S3.

### Overall outcomes

**Figure 2:**
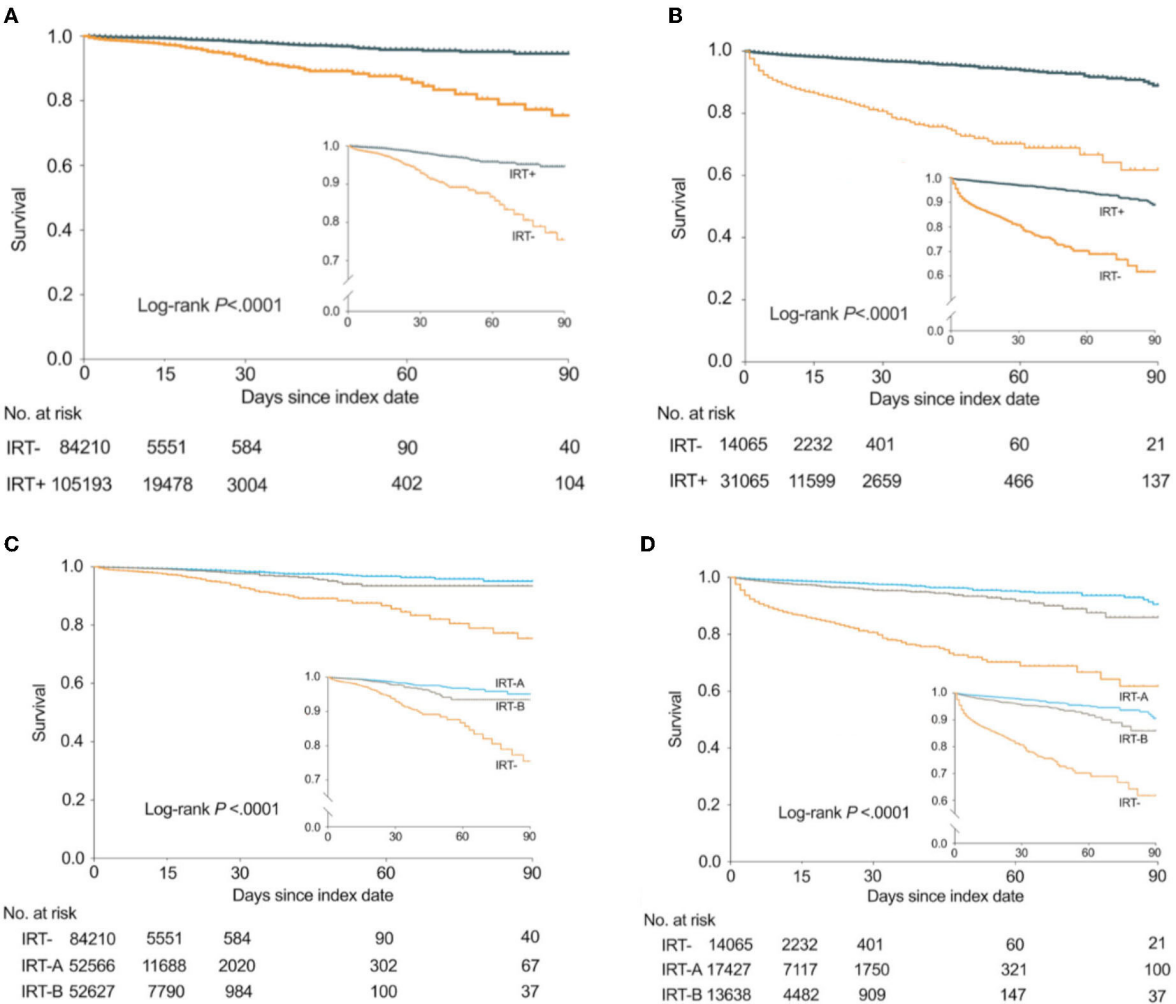
Kaplan-Meier curves showing the probability of survival in the thrombolysis-only group and the EVT group. EVT, endovascular therapy; IRT-A, interventions with PT or OT; IRT-B, interventions without PT/OT. The number of observations was 189,403 in the thrombolysis-only group and 45,130 in the EVT group, for exclusion of missing data on follow-up times. **(A)** In the thrombolysis-only group, the probability of survival between IRT- and IRT+ subgroups. **(B)** In the EVT group, the probability of survival between IRT- and IRT+ subgroups. **(C)** In the thrombolysis-only group, the probability of survival between IRT-, IRT-A, and IRT-B subgroups. **(D)** In the EVT group, the probability of survival between IRT-, IRT-A, and IRT-B subgroups.

In the thrombolysis-only group, the mortality rates in all the patients, IRT- group, and the IRT+ group were 1.4% (2,575/189,519), 2.3% (1,908/84,275), and 0.6% (667/105,244), respectively. The median follow-up times for the IRT- and IRT+ groups were 7 and 10 days, respectively. The Kaplan-Meier curve of survival is shown in Figure 2. The unadjusted Cox model shows that IRT was associated with lower risk of all-cause in-hospital mortality [hazard ratio (HR) 0.2 (95% CI 0.19–0.22); Table 2]. The association remained significant after adjustment [adjusted HR 0.18 (95% CI 0.16–0.2); Table 2]. The times from index date to death were 2 days in the IRT- group and 5 days in the IRT+ group (*p* < 0.0001).

**Table 2:** Cox regression and propensity-matched analyses for IRT and all-cause in-hospital mortality in patients with reperfusion therapy.

	**Unmatched analyses**				**Propensity-matched analyses** ^c^			
	**Unadjusted**		**Adjusted**		**Unadjusted**		**Adjusted**	
	**HR (95% CI)**	***p*** **-value**	**HR (95% CI)**	***p*** **-value**	**HR (95% CI)**	***p*** **-value**	**HR (95% CI)**	***p*** **-value**
**Thrombolysis-only group** ^a^									
IRT-	1 (Reference)	NA	1 (Reference)	NA	1 (Reference)	NA	1 (Reference)	NA
IRT+	0.20 (0.19–0.22)	<0.0001	0.18 (0.16–0.20)	<0.0001	0.25 (0.22–0.29)	<0.0001	0.23 (0.20–0.26)	<0.0001
**EVT group** ^b^									
IRT-	1 (Reference)	NA	1 (Reference)	NA	1 (Reference)	NA	1 (Reference)	NA
IRT+	0.11 (0.10–0.13)	<0.0001	0.13 (0.12–0.15)	<0.0001	0.21 (0.18–0.24)	<0.0001	0.18 (01.5–0.21)	<0.0001

EVT, endovascular therapy; HR, hazard ratio; IRT, inpatient rehabilitation therapy.

a The model adjusted for patient age, hospital level, TOAST, reperfusion time, use of antiplatelet within 48 h, use of statin, NIHSS score at 24 h, NIHSS score change at 24 h, and intracranial hemorrhage, shown in Supplementary Table S12. A total of 159,229 patients were included in the model. Missing data are shown in Supplementary Tables S3, S4.

b The model adjusted for patient age, hospital level, TOAST, reperfusion time, reperfusion status, use of antiplatelet within 48 h, use of statin, NIHSS score at 24 h, NIHSS score change at 24 h, and intracranial hemorrhage, shown in Supplementary Table S13. A total of 31,956 patients were included in the model. Missing data are shown in Supplementary Tables S3, S4.

c Matched on propensity score with age, sex, hospital level, region, mRS at admission, NIHSS score at admission, NIHSS score at 24 h, NIHSS score change at 24 h, TOAST, onset-to-needle time, thrombolytic drugs, use of antiplatelet within 48 h, use of statin, intracranial hemorrhage, and period of COVID-19 in the thrombolysis-only group. Matched on propensity score with age, sex, hospital level, region, mRS at admission, NIHSS score at admission, NIHSS score at 24 h, NIHSS score change at 24 h, TOAST, onset-to-puncture time, reperfusion status, type of EVT (direct EVT or bridging thrombolysis), use of antiplatelet within 48 h, use of statin, intracranial hemorrhage, and period of COVID-19 in the EVT group.

In the EVT group, the median follow-up time in the IRT- and IRT+ groups was 6 and 12 days, respectively. The Kaplan-Meier curve of survival is shown in Figure 2. Table 2 shows the association between IRT and mortality. Patients with IRT showed lower all-cause in-hospital mortality [2 vs. 12.1%; adjusted HR, 0.13 (95% CI 0.12–0.15); overall in-hospital mortality 5.2% (2,341/45,211)]. The median day from index date to death in the IRT+ group (6 days) was longer than that in the IRT- group (2 days) (*p* < 0.0001).

In addition, interventions without PT/OT (IRT-B) also significantly reduced mortality in thrombolysis-only [unadjusted HR 0.22 (95% CI 0.2–0.25)] and EVT [unadjusted HR 0.16 (95% CI 0.14–0.18)], but were inferior to PT/OT (IRT-A) (Figure 2 and Table 3).

**Table 3:** Cox regression models for the IRT subgroups and all-cause in-hospital mortality in patients with reperfusion therapy.

		**Unadjusted-1**		**Unadjusted-2**	
	**Event, *n* (%)**	**HR (95% CI)**	***p*** **-value**	**HR (95% CI)**	***p*** **-value**
**Thrombolysis-only group**					
IRT-	1,908 (2.3)	1 (Reference)	NA	4.46 (3.96–5.02)	<0.0001
IRT-A	318 (0.6)	0.19 (0.16–0.21)	<0.0001	0.82 (0.71–0.96)	0.015
IRT-B	349 (0.7)	0.22 (0.20–0.25)	<0.0001	1 (Reference)	NA
**EVT group**					
IRT-	1,707 (12.1)	1 (Reference)	NA	6.34 (5.65–7.12)	<0.0001
IRT-A	265 (1.5)	0.08 (0.07–0.09)	<0.0001	0.52 (0.44–0.61)	<0.0001
IRT-B	369 (2.7)	0.16 (0.14–0.18)	<0.0001	1 (Reference)	NA

EVT, endovascular therapy; HR, hazard ratio; IRT, inpatient rehabilitation therapy. IRT-A, Interventions with PT or OT; IRT-B, Interventions without PT/OT.

### Propensity-matched analyses

The PSM analyses also showed similar results that IRT was associated with lower in-hospital mortality in both the thrombolysis-only group [adjusted HR 0.23 (95% CI 0.2–0.26)] and the EVT group [adjusted HR 0.18 (95% CI 0.15–0.21)] (Table 2).

### Subgroup analyses

**Figure 3:**
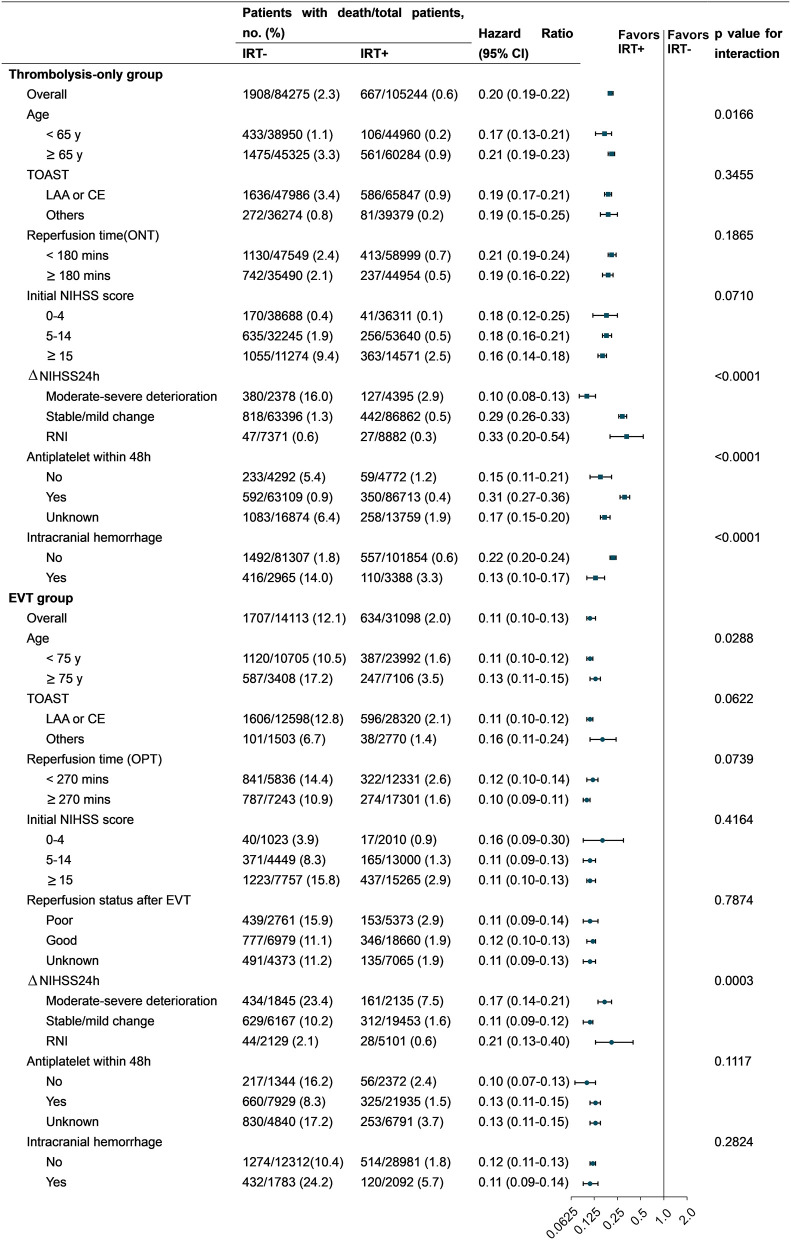
HRs for all-cause in-hospital mortality in subgroup analyses. CE, cardioembolism; EVT, endovascular therapy; IRT, inpatient rehabilitation therapy; LAA, large artery atherosclerosis; NIHSS, National Institutes of Health Stroke Scale; RNI, rapid neurological improvement; ONT, onset-to-needle time; OPT, onset-to-puncture time. Others of TOAST: small artery occlusion/ stroke of other determined cause/stroke of undetermined cause. Forest plots: (1) square for the thrombolysis-only group and (2) round for the EVT group.

The results of the subgroup analyses are presented in Figure 3 and Supplementary Table S8. The reduction in mortality rate with IRT was significant across all major subgroups in both the thrombolysis-only and EVT groups. IRT seemed to be less effective in patients with mild neurologic deficits, but its effect was increased on younger patients. The effect of IRT also varied in different regions of China. However, the associations between IRT and mortality did not vary by TOAST classification and COVID-19 pandemic in both the thrombolysis-only and EVT groups. Finally, significantly higher mortality rates were observed in patients with NIHSS score ≥ 21 and whose proportions of older age and ICH were higher (Supplementary Table S9).

### Sensitivity analyses

In clinical practice, discharge or death within the first day of stroke onset might affect the implementation of IRT and subsequently introduce bias. Therefore, we excluded patients with a follow-up time of 0 day and found that the HRs in the thrombolysis-only and EVT groups were 0.19 (95% CI 0.17– 0.21) and 0.13 (95% CI 0.12– 0.15), respectively. For patients with short hospitalization (follow-up time within 1 week), IRT was still associated with lower mortality risk in the thrombolysis-only group [HR 0.36 (95% CI 0.31–0.41)] and the EVT group [HR 0.27 (95% CI 0.23–0.32)]. In addition, time-dependent Cox regression models confirmed the association between IRT and mortality (Supplementary Tables S10, S11).

## Discussion

This nationwide registry study on patients with ischemic stroke with intravenous thrombolysis or EVT demonstrated that IRT was significantly associated with lower all-cause in-hospital mortality. In-hospital mortality risks were reduced by 80% in the thrombolysis-only group with a median follow-up time of 9 days, and 89% in the EVT group with 11 days. General post-stroke rehabilitation has been shown to be associated with lower mortality risk with volume-dependent effect in patients with ischemic stroke, but these were retrospective studies including patients with and without intravenous thrombolysis (6, 20, 21). Numerous reports demonstrated that r-tPA and EVT improved the functional outcome but did not lower the mortality of the reperfusion group (4, 16, 22). Approximately one in six patients died at 3 months even after timely and effective reperfusion therapy, affected by various risk factors (23). The optimal regimen to further reduce mortality in patients with AIS after reperfusion therapy remains inconclusive. Our prospective registry study suggests that sequential therapy rehabilitation after reperfusion treatment might further reduce in-hospital mortality.

PT and OT were highly recommended for stroke rehabilitation in China and western countries and were widely used (7, 12, 24, 25). As the definition of IRT varied by studies (26), the efficacy of acupuncture, swallowing therapy, and other interventions is controversial (14, 15, 27). Our study found that rehabilitation interventions without PT/OT were still effective in reducing in-hospital mortality although inferior to PT/OT. This gap was of minimal clinical significance. In addition, we speculated that inclusion of interventions other than PT/OT did not compromise the efficacy of IRT in our study.

In the thrombolysis-only group of our study, patients without IRT showed a lower in-hospital mortality risk than those in other countries (2.3 vs. 4.9–11.4%), probably because of younger age and less intracranial hemorrhage cases (23, 28). Another reason is that patients with severer neurologic deficits were transferred from the thrombolysis-only group to the EVT group, thus lowering mortality in the thrombolysis-only group. However, in the EVT group without IRT, the in-hospital mortality rate was similar to that in America (12.1 vs. 12.4%) under similar duration of hospitalization (6 vs. 7 days) (29). A nationwide study from France included ischemic patients with and without intravenous thrombolysis and reported that in-hospital mortality was decreased from 12.6% in general rehabilitation units to 2.6% in neurological rehabilitation units, with a median length of rehabilitation of 36 and 58 days, respectively (20). While 80% of early IRT in our study happened at bedside, which is more feasible and widely used in clinical practice, suggesting that the effect of IRT was not limited by its location.

Rehabilitation in most studies lasted several weeks, and greater volume of IRT predicted lower mortality (21, 26). The efficacy of short-time IRT in mortality remains unknown. The efficacy of IRT in our study was consistent regardless of duration of hospitalization. Moreover, the median duration of hospitalization in our study was similar to that reported in China and other countries (about 1 to 2 weeks) (20, 29, 30). The efficacy of IRT was not influenced by the COVID-19 pandemic. Therefore, the results are not restricted to our patient cohort and may be extrapolated to a larger population of patients with AIS treated with reperfusion therapy after the COVID-19 pandemic.

Severe stroke was proved to be associated with early mortality (31). We noticed imbalances in the baseline clinical data between patients with and without IRT. In the thrombolysis-only group, IRT+ patients had severer neurologic deficit but lower mortality rate compared with IRT- patients, confirming the efficacy of IRT. In the EVT group, IRT+ patients had milder stroke than IRT- patients, but the HR of IRT in the EVT group still showed significance after adjustment for stroke severity. Another concern addressed by our study was that the efficacy of IRT was not influenced by stroke severity but tended to be weak in mild stroke. Unfortunately, in our thrombolysis-only group, 75% of IRT- patients were those with a 24-h NINSS score of 0–4. Neurologists might underestimate the role of IRT, probably because of insufficient evidence (17, 32). We speculated that IRT possibly reduced the risk of deep venous embolism and pneumonia (33, 34), thus lowering mortality. However, the mechanism remained unknown, warranting further studies.

Another concern was that the imbalance in the baseline data might generate a bias to the association between IRT and in-hospital mortality. Some researchers questioned that IRT was performed on patients with stable status, but about half of patients with severe stroke received IRT in our study. Moreover, the results of Cox regression, propensity score matching, and stratification analyses all supported the association between IRT and mortality. The impact of imbalanced baseline date might be limited. Finally, we noticed that the main result should be interpreted cautiously for inherent bias.

ICH is the most feared and major complication of thrombolysis and EVT (4, 5, 23), and is associated with mortality. However, the efficacy of IRT in patients with ICH was not inferior to that in those without ICH. IRT might be a reasonable choice to reduce mortality caused by ICH.

Longer median follow-up time was reported in the IRT+ group. The imbalance of follow-up times could lead to a spurious difference in death between the IRT- and IRT+ groups. Theoretically, more deaths were expected to be observed the in IRT+ group for their longer follow-up days in both the thrombolysis-only and EVT groups. However, less deaths were observed in the IRT+ group, thus further demonstrating the efficacy of IRT.

## Limitation

This study has several limitations. First, many factors were associated with mortality (23, 31, 35), but some comorbidities and complications were not documented in our registry data, such as pneumonia and congestive heart failure, which might generate bias. Second, the intensity, duration, and start time of IRT were not included in our study, thus the quality of IRT might be heterogeneous (32). To rectify this limitation, we listed the contents of IRT in the Supplementary material. Third, the endpoint of our study was in-hospital mortality and suboptimal to mortality at 90 days after stroke. Previous studies showed that more than half of 90 day-follow-up death cases occurred within 7 days after stroke (36, 37). As the median durations of hospitalization in our study were between 7 and 14 days and the HRs of IRT were significant at 90 days in the Kaplan-Meier curves, we regarded all-cause in-hospital mortality as a reliable intermediate endpoint, probably in line with the 90-day mortality. Fourth, the causes of mortality were undocumented, the mechanism by which IRT reduced death remained masked. Fifth, since our database did not register patients with AIS without reperfusion therapy, our conclusion could not be extrapolated to those patients. Finally, there might be unmeasured confounders, which may influence IRT and outcomes, although the Cox regression model adjusted for multiple patient- and hospital-level characteristics.

## Conclusions

Among patients with ischemic stroke treated with intravenous thrombolysis or endovascular therapy, sequential therapy by rehabilitation was associated with lower all-cause in-hospital mortality. These findings suggest the necessity of promoting inpatient rehabilitation therapy after reperfusion in patients with ischemic stroke.

## Data availability statement

The data supporting this study are available from the corresponding author upon reasonable request.

## Ethics statement

The studies involving human participants were reviewed and approved by the Ethics Committee of Peking Union Medical College Hospital. Written informed consent from the participants' legal guardian/next of kin was not required to participate in this study in accordance with the national legislation and the institutional requirements.

## Author contributions

SL: substantial contribution to the conception or design of the study, acquisition, analysis, interpretation of data for the study, and drafting of the manuscript. SF: analysis or interpretation of data for the study. DZ: analysis of the data for the study. YL: interpretation of the data for the study. LW: substantial contribution to the conception or design of the study and critical revision of the article for important intellectual content. BP: substantial contributions to the conception or design of the study, critical revision of the study for important intellectual content, and final approval of the version to be published. All authors contributed to the article and approved the submitted version.

## Funding

This study was funded by the National Key Research and Development Program of China (2016 YFC0901004).

## Conflict of interest

The authors declare that the research was conducted in the absence of any commercial or financial relationships that could be construed as a potential conflict of interest.

## Publisher's note

All claims expressed in this article are solely those of the authors and do not necessarily represent those of their affiliated organizations, or those of the publisher, the editors and the reviewers. Any product that may be evaluated in this article, or claim that may be made by its manufacturer, is not guaranteed or endorsed by the publisher.
