# Supplementary material for: Association between rehabilitation after reperfusion treatment and in-hospital mortality: Results from a national registry study
Source: Front Neurol. 2022 Sep 14;13:949669. doi: 10.3389/fneur.2022.949669 (PMC9515317; doi:10.3389/fneur.2022.949669)

Supplementary Online Content

TableS1. Definitions of key variables
TableS2. Missing data of primary outcome in thrombolysis-only and EVT groups.
TableS3. Missing data of variables in thrombolysis-only and EVT groups.
TableS4. Missing data of follow up time in thrombolysis-only and EVT groups
TableS5. Demographic and clinical characteristics of patients with thrombolysis-only and endovascular therapy
TableS6. The structure and contents of IRT in thrombolysis-only and EVT groups
TableS7. Demographic and clinical characteristics of patients with thrombolysis-only and endovascular therapy after PSM
TableS8. HRs for all-cause in-hospital mortality in subgroup analyses in patients with only thrombolysis and EVT
TableS9. Clinical characteristics of patients with initial NIHSS score ≥ 21
TableS10. Sensitivity analyses of Cox regression models in patients with only thrombolysis
TableS11. Sensitivity analyses of Cox regression models in patients with EVT
TableS12. Confounders of Cox regression model in thrombolysis-only group
TableS13. Confounders of Cox regression model in EVT group
FigureS1. Data source of our study
FigureS2. The distribution of propensity scores in unmatched and matched patients in thrombolysis-only group
FigureS3. The distribution of propensity scores in unmatched and matched patients in EVT group

TableS1. Definitions of key variables [a]

Variable	Definition	Note
Drugs of intravenous thrombolysis	(1) Standard r-tPA (0.85-0.95mg/kg);<br>(2) Suboptimal r-tPA (<0.85mg/kg or > 0.95mg/kg);<br>(3) Unknown r-tPA (Without documented r-tPA dosage per kg);<br>(4) Other drugs: including urokinase, anistreplase, reteplase, tenecteplase, and recombinant human pro-urokinase.	NA
△NIHSS24h	△NIHSS24h=initial NIHSS score–NIHSS score at 24 hours after reperfusion procedure.	NA
Change of NIHSS score at 24 hour	(1) Moderate-severe deterioration, △NIHSS24h ≤-4;<br>(2) Mild deterioration, -3≤△NIHSS24h≤-1;<br>(3) Stable status, △NIHSS24h=0;<br>(4) Mild improvement, 1≤△NIHSS24h≤7;<br>(5) Rapid neurological improvement (RNI), △NIHSS24h≥ 8.	NA
Change of NIHSS score at 24 hour	(1)Severe deterioration (△NIHSS24h ≤-9);<br>(2)Non-severe deterioration (△NIHSS24h ≥ -8).	Another subtypes of △NIHSS24h.
Reperfusion status after EVT	(1) Poor: modified TICI score of 0, 1, or 2a;<br>(2) Good: modified TICI score of 2b or 3;<br>(3)Unknown: without documented modified TICI score.[1]	NA
Procedure of EVT	(1) Bridging thrombolysis (BT), EVT with intravenous thrombolysis;<br>(2) Direct EVT: EVT without intravenous thrombolysis.	NA
Hospital levels: 3 levels	(1) class A: Tertiary A hospitals;<br>(2) class B: tertiary-B or tertiary-C or undetermined tertiary hospitals;<br>(3) class C, secondary and primary hospitals.	All hospitals in China were graded by the National Health Commission with standard criteria as primary, secondary, and tertiary hospitals, and A-B-C sub-levels in each group, mainly based on the quality of medical care and management. Tertiary A represents the highest level in this grading system.

Covid19 pandemic: 2 periods	(1)"Before Covid19 pandemic" covered date from January 1, 2019 to January 22, 2020;<br>(2)"During Covid19 pandemic" covered date from January 23, 2020 to June 30, 2020.	NA
Intracranial hemorrhage	It was reported after confirmation of computed tomography results and neurological findings, including symptomatic and asymptomatic events.	NA

[a] Details of variables have been published elsewhere.[2,3]

1. Berkhemer OA, Fransen PS, Beumer D, et al. A randomized trial of intraarterial treatment for acute ischemic stroke. *N Engl J Med*. Jan 1 2015;372(1):11-20. doi:10.1056/NEJMoa1411587

2. Chao BH, Yan F, Hua Y, Liu JM, Yang Y, Ji XM, Peng B, Zhao GG, Wang YJ, Kang DZ, et al. Stroke prevention and control system in China: CSPPC-Stroke Program. Int J Stroke. 2021;16:265-272. doi: 10.1177/1747493020913557

3. Shen Y, Chao BH, Cao L, Tu WJ, Wang LD. Stroke Center Care and Outcome: Results from the CSPPC Stroke Program. Transl Stroke Res. 2020;11:377-386. doi: 10.1007/s12975-019-00727-6.

TableS2. Missing data of primary outcome in thrombolysis-only and EVT groups

a. Thrombolysis-only group

	Overall, n(%)	IRT-, n (%)	IRT+, n (%)	P value
With outcome	189519 (99.7)	84275 (99.7)	105244 (99.6)	<0.0001
Without outcome	534 (0.3)	175 (0.2)	359 (0.3)	

b. EVT group

	Overall, n(%)	IRT-, n (%)	IRT+, n (%)	P value
With outcome	45211 (99.7)	14113 (99.7)	31098 (99.5)	0.0011
Without outcome	196 (0.3)	40 (0.3)	156 (0.5)	

TableS3. Missing data of variables in thrombolysis-only and EVT groups

	Thrombolysis-only group		EVT group	
	Number	Percentage (%)	Number	Percentage (%)
Age	0	0	0	0
Sex	5	0	1	0
Nationality	0	0	0	0
Region	0	0	0	0
Hospital level	29	0.02	1	0
Systolic pressure at admission	38	0.03	8	0.02
BMI	12667	6.5	4216	9.3
TOAST	33	0.02	20	0.04
Reperfusion time	2527	1.3	2500	5.5
Thrombolysis drug	0	0	-	-
mTICI after EVT	-	-	0	0
Initial NIHSS score	1790	0.9	1707	3.8
Initial mRS	31947	16.9	6702	14.8
NIHSS score at 24h	15636	8.3	8109	17.9
△NIHSS24h	16235	8.6	8732	19.3
Intracranial hemorrhage	5	0	43	0.1
Gastrointestinal hemorrhage	5	0	43	0.1
Antiplatelet therapy	0	0	0	0
Statin therapy	6912	3.6	2880	6.4
Covid19 pandemic	0	0	0	0
Duration of follow up	116	0.1	81	0.2

Abbreviations: BMI, body mass index; EVT, Endovascular therapy;

TableS4. Missing data of follow up time in thrombolysis-only and EVT groups

a. Thrombolysis-only group

	IRT-, n (%)	IRT+, n (%)	P value
With follow up time	84210 (99.92)	105193 (99.95)	0.0122
Without follow up time	65 (0.08)	51 (0.05)	

b. Patient with death in thrombolysis-only group

	IRT-, n (%)	IRT+, n (%)	P value
With follow up time	1866 (97.8)	640 (96.0)	0.011
Without follow up time	42 (2.2)	27 (4.0)	

c. EVT group

	IRT-, n (%)	IRT+, n (%)	P value
With follow up time	14065 (99.7)	31065 (99.9)	<0.0001
Without follow up time	48 (0.3)	33 (0.1)	

d. Patient with death in EVT group

	IRT-, n (%)	IRT+, n (%)	P value
With follow up time	1667 (97.7)	609 (96.1)	0.0363
Without follow up time	40 (2.3)	25 (3.9)	

TableS5. Demographic and clinical characteristics of patients with thrombolysis-only and endovascular therapy

	Thrombolysis-only group [a]				Endovascular therapy group [a]			
	Overall	Without IRT	With IRT	P value	Overall	Without IRT	With IRT	P value
Patients	189519	84275 (44.5)	105244 (55.5)		45211	14113 (31.2)	31098 (68.8)	
Age group, y				<0.0001				0.0002
14-40	4037 (2.1)	2015 (2.4)	20242 (1.9)		1501 (3.3)	431 (3.1)	1070 (3.4)	
41-60	57111 (30.1)	26646 (31.6)	30465 (29.0)		13426 (29.7)	4096 (29.0)	9330 (30.0)	
61-70	61303 (32.3)	27369 (32.5)	33934 (32.2)		14489 (32.1)	4528 (32.1)	9961 (32.0)	
71-80	48173 (25.4)	20586 (24.4)	27587 (26.2)		11649 (25.8)	3650 (25.8)	7999 (25.7)	
81-99	18895 (10.0)	7659 (9.1)	11236 (10.7)		4146 (9.2)	1408 (10.0)	2738 (8.8)	
Nationality				0.1355				0.0661
Han	183902 (97.0)	81832 (97.1)	102070 (97.0)		44044 (97.4)	13720 (97.2)	30324 (97.5)	
Minority	5617 (3.0)	2443 (2.9)	3174 (3.0)		1167 (2.6)	393 (2.8)	774 (2.5)	
Region				<0.0001				<0.0001
Northeast	20459 (10.8)	11707 (13.9)	8752 (8.3)		3665 (8.1)	1553 (11.0)	2112 (6.8)	
North	38937 (20.6)	20493 (24.3)	18444 (17.5)		4372 (9.7)	1797 (12.7)	2575 (8.3)	
East	53888 (28.4)	22636 (26.9)	31252 (29.7)		15583 (34.5)	4910 (34.8)	10673 (34.2)	
Central	33015 (17.4)	13754 (16.3)	19261 (18.3)		6485 (14.3)	1927 (13.7)	4558 (14.7)	
South	131080 (6.9)	4195 (5.0)	8885 (8.4)		7576 (16.8)	1855 (13.1)	5721 (18.4)	
Southwest	21398 (11.3)	7909 (9.4)	13489 (12.8)		4993 (11.0)	1324 (9.4)	3669 (11.8)	
Northwest	8742 (4.6)	3581 (4.2)	5161 (4.9)		2537 (5.6)	747 (5.3)	1790 (5.8)	
Systolic pressure at admission (mmHg)	152 (138-168)	151 (137-167)	153 (139-168)	<0.0001	148 (132-165)	148 (131-165)	148 (132-164)	00.2413
Systolic pressure at admission (mmHg)				<0.0001				0.0076
< 160	115064 (60.7)	52190 (61.9)	62874 (59.7)		30531 (67.5)	9407 (66.7)	21124 (67.9)	
≥ 160	74417 (39.3)	32066 (38.1)	42351 (40.3)		14672 (32.5)	4703 (33.3)	9969 (32.1)	

BMI, Kg/m^2	23.7 (21.4-25.9)	23.7 (21.5-26.0)	23.5 (21.3-25.7)	<0.0001	23.8 (22.0-25.7)	23.9 (22.0-25.8)	23.7 (21.9-25.7)	<0.0001
BMI group, Kg/m^2				<0.0001				<0.0001
<18.5	11070 (6.3)	4704 (6.0)	6366 (6.5)		1416 (3.5)	460 (3.6)	956 (3.4)	
18.5-	84520 (47.8)	36535 (46.7)	47985 (48.7)		20259 (49.4)	6006 (47.4)	14253 (50.3)	
24-	63245 (35.8)	28434 (36.4)	34811 (35.3)		15948 (38.9)	5129 (40.5)	10819 (38.2)	
28-	18017 (10.2)	8548 (10.9)	9469 (9.6)		3372 (8.2)	1069 (8.4)	2303 (8.1)	
Reperfusion time, mins [b]				0.0311				<0.0001
<60/120	7210 (3.9)	3287 (4.0)	3923 (3.8)		2417 (5.7)	808 (6.2)	1609 (5.4)	
60-119/120-269	39793 (21.3)	17602 (21.2)	22191 (21.4)		15750 (36.9)	5028 (38.4)	10722 (36.2)	
120-179/270-359	59545 (31.8)	26660 (32.1)	32885 (31.6)		9507 (22.3)	2959 (22.6)	6548 (22.1)	
180-269/360-959	61458 (32.9)	27133 (32.7)	34325 (33.0)		13601 (31.8)	3924 (30.0)	9677 (32.7)	
270-360/960-1440	18986 (10.2)	8357 (10.1)	10629 (10.2)		1436 (3.4)	360 (2.8)	1076 (3.6)	
Thrombolysis drug				<0.0001				…
Standard r-tPA	75934 (40.1)	33008 (39.2)	42926 (40.8)		…	…	…	
Suboptimal r-tPA	56424 (29.8)	25074 (29.7)	31350 (29.8)		…	…	…	
Unknown r-tPA	8276 (4.4)	3884 (4.6)	4392 (4.2)		…	…	…	
Other drugs	48885 (25.8)	22309 (26.5)	26576 (25.3)					
Reperfusion status after EVT [c]			…					<0.0001
Poor	…	…	…		8134 (18.0)	2761 (19.6)	5373 (17.3)	
Good	…	…	…		25639 (56.7)	6979 (49.4)	18660 (60.0)	
Unknown	…	…	…		11438 (25.3)	4373 (31.0)	7065 (22.7)	
Initial mRS				<0.0001				<0.0001
0-2	100411 (63.7)	45348 (67.2)	55063 (61.1)		19599 (50.9)	5553 (49.1)	14046 (51.7)	
3-5	57161 (36.3)	22090 (32.8)	35071 (38.9)		18910 (49.1)	5761 (50.9)	13149 (48.3)	
NIHSS score at 24h				<0.0001				<0.0001

0-4	112327 (64.6)	55424 (75.4)	56903 (56.7)		7793 (21.0)	2768 (27.0)	5025 (18.7)	
5-9	31240 (18.0)	8274 (11.3)	22966 (22.9)		7659 (20.6)	1333 (13.0)	6326 (23.6)	
10-14	15895 (9.1)	3843 (5.2)	12052 (12.0)		7925 (21.4)	1293 (12.6)	6632 (24.7)	
15-20	8729 (5.0)	2954 (4.0)	5775 (5.7)		6911 (18.6)	1642 (16.0)	5269 (19.6)	
≥ 21	5692 (3.3)	2965 (4.0)	2727 (2.7)		6814 (18.4)	3222 (31.4)	3592 (13.4)	
Covid19 pandemic				0.0313				0.1152
Before	173154 (91.4)	76867 (91.2)	96287 (91.5)		41164 (91.1)	12894 (91.4)	28270 (90.9)	
During	16365 (8.6)	7408 (8.8)	8957 (8.5)		4047 (8.9)	1219 (8.6)	2828 (9.1)	
Duration of follow up, d				<0.0001				<0.0001
0-6	63150 (33.3)	37030 (44.0)	26120 (24.8)		12921 (28.6)	7040 (50.1)	5881 (18.9)	
7-20	116339 (61.4)	45463 (54.0)	70876 (67.4)		24983 (55.4)	5966 (42.4)	19071 (61.2)	
21-	9916 (5.2)	1717 (2.0)	8199 (7.8)		7226 (16.0)	1059 (7.5)	6167 (19.9)	

Abbreviations: BMI, body mass index; IRT, inpatient rehabilitation therapy.

[a] Categorical variables: Frequencies and percentages; Continuous variables with non-normal distributions: Median and interquartile range (IQR).

[b] Left column indicates onset-to-needle time (ONT) in patients with only intravenous thrombolysis; Right column indicates onset-to-puncture time (OPT) in patients with endovascular therapy.

[c] Reperfusion status: classed according to modified TICI score.

Table6. The structure and contents of IRT in thrombolysis-only and EVT groups

Items	Thrombolysis-only group, n(%)	EVT group, n(%)
Approaches of IRT		
Acupuncture/massage	66884 (35.3)	21931 (48.5)
Physical therapy	50528 (26.7)	16871 (37.3)
Occupational therapy	20469 (10.8)	6889 (15.2)
Speech therapy	24306 (12.9)	8953 (19.8)
Other interventions of rehabilitation [a]	40908 (21.6)	12683 (28.1)
Model of IRT [b]		
Without IRT	84275 (44.5)	14113 (31.2)
Single intervention	53239 (28.1)	13048 (28.9)
Multimodal intervention	52005 (27.4)	18050 (39.9)
Rehabilitation locations [c]		
Only bedside	83534 (80.5)	24401 (79.2)
Inpatient rehabilitation center	20288 (19.5)	6394 (20.8)

Abbreviations: EVT, endovascular therapy; IRT, inpatient rehabilitation therapy.

[a] Other interventions of rehabilitation included cognitive training, swallowing therapy, psychotherapy, or physiotherapy based on traditional Chinese medicine.

[b] Single intervention for patients with only one approach; Multimodal intervention for patients with 2-5 approaches.

[c] Number and percentages was for proportions of locations and calculated in patients with IRT. Inpatient rehabilitation center included patients trained in inpatient rehabilitation center, with our without bedside rehabilitation therapy

TableS7. Demographic and clinical characteristics of patients with thrombolysis-only and endovascular therapy after PSM

	Thrombolysis-only group			Endovascular therapy group		
	Without IRT	With IRT	P value	Without IRT	With IRT	P value
	(N= 56923)	(N= 56923)		(N=7710)	(N=7710)	
Age, y	66 (57-74)	66 (57-74)	0.3511	66 (56-74)	66 (57-74)	0.5909
Age group, y			0.8403			0.4993
14-40	1257 (2.2)	1219 (2.1)		215 (3.3)	277 (3.6)	
41-60	17647 (31.0)	17519 (30.8)		2308 (29.9)	2232 (29.0)	
61-70	18687 (32.8)	18761 (33.0)		2469 (32.0)	2500 (32.4)	
71-80	14035 (24.7)	14110 (24.8)		1943 (25.2)	1981 (26.0)	
81-99	5297 (9.3)	5314 (9.3)		739 (9.6)	720 (9.3)	
Sex, Male	36816 (64.7)	36855 (64.8)	0.8098	4910 (63.7)	4924 (63.9)	0.8145
Region			0.4699			0.5860
Northeast	6489 (11.4)	6330 (11.1)		878 (11.4)	896 (11.6)	
North	12696 (22.3)	12809 (22.5)		904 (11.7)	878 (11.4)	
East	16344 (28.7)	16282 (28.6)		2528 (32.8)	2434 (31.6)	
Central	10449 (18.4)	10364 (18.2)		1196 (15.5)	1211 (15.7)	
South	3000 (5.3)	3119 (5.5)		1029 (13.4)	1050 (13.6)	
Southwest	5454 (9.6)	5507 (9.7)		758 (9.8)	812 (10.5)	
Northwest	2491 (4.4)	2512 (4.4)		417 (5.4)	429 (5.6)	
Hospital level			0.8745			0.7190
Class A	29224 (51.3)	29275 (51.4)		6607 (85.7)	6642 (86.2)	
Class B	8151 (14.3)	8091 (14.2)		622 (8.1)	601 (7.8)	
Class C	19548 (34.3)	19557 (34.4)		481 (6.2)	467 (6.1)	
TOAST			0.6706			0.6179
LAA	26624 (46.8)	26872 (47.2)		4552 (59.0)	4603 (59.7)	

CE	5627 (9.9)	5614 (9.9)		2432 (31.5)	2429 (31.5)	
SAO	21924 (38.5)	21723 (38.2)		322 (4.2)	316 (4.1)	
SOC	415 (0.7)	416 (0.7)		124 (1.6)	112 (1.5)	
SUC	2333 (4.1)	2298 (4.0)		280 (3.6)	250 (3.2)	
BMI, Kg/m^2	23.7 (21.5-26.0)	23.7 (21.5-26.0)	0.9744	23.9 (22.0-25.8)	23.8 (22.0-25.8)	0.6432
Reperfusion time, mins	165 (119-220)	165 (118-220)	0.7454	285 (200-402)	289 (195-405)	0.6873
Reperfusion time, mins [b]			0.0859			0.4978
<60/120	2260 (4.0)	2194 (3.9)		502 (6.5)	466 (6.0)	
60-119/120-269	12138 (21.3)	12381 (21.8)		2944 (38.2)	3007 (39.0)	
120-179/270-359	18272 (32.1)	17975 (31.6)		1705 (22.1)	1687 (21.9)	
180-269/360-959	18642 (32.8)	18599 (32.7)		2332 (30.3)	2346 (30.4)	
270-360/960-1440	5611 (9.9)	5773 (10.1)		227 (2.9)	204 (2.7)	
Thrombolysis drug			0.9623			
Standard r-tPA	23944 (42.1)	23857 (41.9)		…	…	…
Suboptimal r-tPA	17994 (31.6)	18039 (31.7)		…	…	…
Unknown r-tPA	476 (0.8)	481 (0.9)		…	…	…
Other drugs	14509 (25.5)	14546 (25.6)		…	…	…
Reperfusion status after EVT			...			0.9835
Poor	…	…		1676 (21.7)	1678 (21.8)	
Good	…	…		4510 (58.5)	4500 (58.4)	
Unknown				1524 (19.8)	1532 (19.9)	
EVT type			...			0.0772
BT	…	…		2384 (30.9)	2486 (32.2)	
Direct EVT	…	…		5326 (69.1)	5224 (67.8)	
Initial NIHSS score	5 (3-9)	5 (3-9)	<0.0001	15 (10-21)	15 (10-21)	0.1356

Initial NIHSS score			0.6766			0.9418
0-4	24753 (47.0)	26547 (46.6)		656 (8.5)	659 (8.6)	
5-9	16802 (29.5)	16847 (29.6)		1064 (13.8)	1095 (14.2)	
10-14	6920 (12.2)	7061 (12.4)		1780 (23.1)	1778 (23.1)	
15-20	4019 (7.1)	4025 (7.1)		2152 (27.9)	2156 (28.0)	
≥21	2429 (4.3)	2443 (4.3)		2058 (26.7)	2022 (26.2)	
Initial mRS	1 (0-3)	1 (0-3)	0.0064	3 (0-4)	3 (0-4)	0.0133
NIHSS score at 24h	2 (0-5)	2 (1-5)	<0.0001	12 (4-21)	12 (4-21)	0.9519
NIHSS score at 24h			0.8426			0.7536
0-4	41941 (73.7)	41806 (73.4)		2188 (28.4)	2213 (28.7)	
5-9	7200 (12.7)	7286 (12.8)		1119 (14.5)	1158 (15.0)	
10-14	3340 (5.9)	3402 (6.0)		1052 (13.6)	1068 (13.9)	
15-20	2452 (4.3)	2459 (4.3)		1342 (17.4)	1313 (17.0)	
≥ 21	1990 (3.5)	1970 (3.5)		2009 (26.1)	1958 (25.4)	
△NIHSS24h			0.3126			0.8534
Moderate-Severe deterioration	1857 (3.3)	1760 (3.1)		1154 (15.0)	1126 (14.6)	
Mild deterioration	1909 (3.4)	1953 (3.4)		468 (6.1)	469 (6.1)	
Stable	12896 (22.7)	13084 (23.0)		1936 (25.1)	1905 (24.7)	
Mild improvement	34668 (60.9)	34526 (60.7)		2368 (30.7)	2428 (31.5)	
RNI	5593 (9.8)	5600 (9.8)		1784 (23.1)	1782 (23.1)	
Antiplatelet within 48h			0.7808			0.5895
No	2566 (4.5)	2573 (4.5)		711 (9.2)	697 (9.0)	
Yes	48268 (84.8)	48334 (84.9)		5256 (68.2)	5315 (68.9)	
Unknown	6089 (10.7)	6016 (10.6)		1743 (22.6)	1698 (22.0)	
Statin			0.0051			0.8268

No	2666 (4.7)	2470 (4.3)		1251 (16.2)	1241 (16.1)	
Yes	54257 (95.3)	54453 (95.7)		6459 (83.8)	6469 (83.9)	
ICH			0.9434			0.8486
No	55293 (97.1)	55289 (97.1)		6962 (90.3)	6969 (90.4)	
Yes	1630 (2.9)	1634 (2.9)		748 (9.7)	741 (9.6)	
GIH			0.4887			1.0000
No	56574 (99.4)	56592 (99.4)		7681 (99.6)	7681 (99.6)	
Yes	349 (0.6)	331 (0.6)		29 (0.4)	29 (0.4)	
Duration of follow up, d	8 (5-11)	9 (6-13)	<0.0001	8 (3-12)	12 (7-18)	<0.0001
Covid19 pandemic			0.6163			0.9510
Before	52363 (92.0)	52317 (91.9)		7138 (92.6)	7136 (92.6)	
During	4560 (8.0)	4606 (8.1)		572 (7.4)	574 (7.4)	

Abbreviations: BT, bridging thrombolysis; CE, cardioembolism; DET, direct endovascular therapy; GIH, gastrointestinal hemorrhage; ICH, intracranial hemorrhage; IRT, inpatient rehabilitation therapy; LAA, large artery atherosclerosis; mRS, modified ranking score; NIHSS, National Institutes of Health Stroke Scale; RNI, rapid neurological improvement; SAO, small artery occlusion; SOC, stroke of other determined cause; SUC, stroke of undetermined cause.

[a] Categorical variables: Frequencies and percentages; Continuous variables with non-normal distributions: Median and interquartile range (IQR).

[b] Left column indicates onset-to-needle time (ONT) in patients with only intravenous thrombolysis; Right column indicates onset-to-puncture time (OPT) in patients with endovascular therapy.

TableS8. HRs for all-cause in-hospital mortality in subgroup analyses in patients with only thrombolysis and EVT

	Patients with events/total patients, no. (%)		Hazard Ratio (95% CI)	P value
	IRT-	IRT+		
Thrombolysis-only group				
Overall	1908/84275 (2.3)	667/105244(0.6)	0.20 (0.19-0.22)	
Sex				0.6123
Male	1094/54479 (2.0)	371/67320 (0.6)	0.20 (0.18-0.22)	
Female	814/29793 (2.7)	296/37922 (0.8)	0.21 (0.18-0.24)	
Systolic Pressure at admission				0.003
< 160 mmHg	1036/52190 (2.0)	382/62874 (0.6)	0.23 (0.20-0.26)	
≥ 160 mmHg	871/32066 (2.7)	284/42351 (0.7)	0.18 (0.15-0.20)	
Region				0.0320
Northeast	477/11707 (4.1)	67/8752 (0.8)	0.15 (0.11-0.19)	
North	314/20493 (1.5)	72/18444 (0.4)	0.19 (0.14-0.24)	
East	438/22636 (1.9)	162/31252 (0.5)	0.19 (0.16-0.23)	
Central	258/13754 (1.9)	119/19261 (0.6)	0.24 (0.19-0.30)	
South	106/4195 (2.5)	80/8885 (0.9)	0.24 (0.18-0.32)	
Southwest	211/7909 (2.7)	130/13489 (1.0)	0.24 (0.19-0.30)	
Northwest	104/3581 (2.9)	37/5161 (0.7)	0.17 (0.12-0.25)	
Hospital level				0.2876
Class A	172/40838 (3.1)	487/58268 (0.8)	0.20 (0.18-0.23)	
Class B	222/12101 (1.8)	73/14405 (0.5)	0.20 (0.15-0.26)	
Class C	414/31312 (1.3)	107/32566 (0.3)	0.17 (0.14-0.22)	
TOAST				0.5855
LAA	1042/39231 (2.7)	357/53957 (0.7)	0.18 (0.16-0.20)	
CE	594/8755 (6.8)	229/11890 (1.9)	0.19 (0.16-0.22)	
SAO	133/30970 (0.4)	47/35014 (0.1)	0.21 (0.14-0.29)	
SOC	20/689 (2.9)	5/780 (0.6)	0.17 (0.06-0.45)	
SUC	119/4615 (2.6)	29/3585 (0.8)	0.22 (0.15-0.34)	
Initial NIHSS score				0.2392
0-4	170/38688 (0.4)	41/36311 (0.1)	0.18 (0.12-0.25)	
5-9	260/22614 (1.2)	103/34863 (0.3)	0.19 (0.15-0.25)	
10-14	375/10631 (3.5)	153/18777 (0.8)	0.15 (0.13-0.19)	
15-20	512/6702 (7.6)	190/9912 (1.9)	0.15 (0.13-0.18)	
21-	543/4752 (11.9)	173/4695 (3.7)	0.18 (0.15-0.21)	
Initial mRS				0.0023
0-2	629/45348 (1.4)	231/55063 (0.4)	0.22 (0.19-0.26)	
3-5	884/22090 (4.0)	351/35071 (1.0)	0.18 (0.16-0.20)	
NIHSS score at 24h				<0.0001
0-4	88/55424 (0.2)	52/56903 (0.1)	0.43 (0.30-0.61)	
5-9	110/8274 (1.3)	71/22966 (0.3)	0.16 (0.12-0.22)	
10-20	481/6797 (7.1)	274/17827 (1.5)	0.14 (0.12-0.16)	

21-	572/2965 (19.3)	200/2727 (7.3)	0.20 (0.17-0.24)	
Thrombolysis drug				0.0003
Standard r-tPA	827/33008 (2.5)	266/42926 (0.6)	0.18 (0.16-0.21)	
Suboptimal r-tPA	602/25074 (2.4)	267/31350 (0.9)	0.25 (0.22-0.30)	
Unknown r-tPA	103/3884 (2.7)	31/4392 (0.7)	0.19 (0.13-0.29)	
Other drugs	376/22309 (1.7)	103/26576 (0.4)	0.16 (0.13-0.21)	
Statin				<0.0001
No	745/8429 (8.8)	110/3602 (3.1)	0.19 (0.15-0.23)	
Yes	1057/72841 (1.5)	521/97735 (0.5)	0.27 (0.24-0.30)	
Covid19 pandemic				
Before	1680/76867 (2.2)	592/96287 (0.6)	0.21 (0.19-0.23)	0.6623
During	228/7408 (3.1)	75/8957 (0.8)	0.19 (0.15-0.25)	
EVT group				
Sex				0.5031
Male	1027/8854 (11.6)	372/20111 (1.9)	0.11 (0.10-0.12)	
Female	680/5259 (12.9)	262/10986 (2.4)	0.12 (0.10-0.14)	
Systolic Pressure at admission				0.1563
< 160 mmHg	1041/9407 (11.1)	370/21124 (1.8)	0.11 (0.09-0.12)	
≥ 160 mmHg	666/4703 (14.2)	263/9969 (2.6)	0.12 (0.11-0.14)	
Region				0.0020
Northeast	343/1553 (22.1)	78/2112 (3.7)	0.12 (0.09-0.15)	
North	172/1797 (9.6)	45/2575 (1.8)	0.15 (0.11-0.21)	
East	438/4910 (8.9)	156/10673 (1.5)	0.10 (0.09-0.13)	
Central	208/1927 (10.8)	94/4558 (2.1)	0.13 (0.10-0.17)	
South	263/1855 (14.2)	117/5721 (2.1)	0.09 (0.07-0.11)	
Southwest	144/1324 (10.9)	106/3669 (2.9)	0.17 (0.13-0.22)	
Northwest	139/747 (18.6)	38/1790 (2.1)	0.08 (0.06-0.11)	
Hospital level				0.1164
Class A	1492/11890 (12.6)	573/26777 (2.1)	0.12 (0.11-0.13)	
Class B	138/1275 (10.8)	47/2820 (1.7)	0.09 (0.06-0.13)	
Class C	77/947 (8.1)	14/1501 (0.9)	0.07 (0.04-0.13)	
TOAST				0.0383
LAA	991/8374 (11.8)	346/19375 (1.8)	0.10 (0.09-0.12)	
CE	615/4224 (14.6)	250/8945 (2.8)	0.12 (0.10-0.14)	
SAO	29/569 (5.1)	10/1425 (0.7)	0.12 (0.06-0.24)	
SOC	22/235 (9.4)	10/475 (2.1)	0.16 (0.08-0.35)	
SUC	50/699 (7.2)	18/870 (2.1)	0.22 (0.13-0.38)	
EVT type				0.0395
BT	564/4033 (14.0)	197/9157 (2.2)	0.10 (0.08-0.12)	
Direct EVT	1143/10080 (11.3)	437/21941 (2.0)	0.12 (0.11-0.14)	
Initial NIHSS score				0.6347
0-4	40/1023 (3.9)	17/2010 (0.9)	0.16 (0.09-0.30)	
5-9	83/1560 (5.3)	43/4558 (0.9)	0.12 (0.08-0.18)	

10-14	288/2889 (10.0)	122/8442 (1.5)	0.11 (0.08-0.13)	
15-20	519/3803 (13.7)	215/9130 (2.4)	0.11 (0.09-0.13)	
21-	704/3954 (17.8)	222/6135 (3.6)	0.12 (0.10-0.14)	
Initial mRS				0.5064
0-2	644/5553 (11.6)	272/14046 (1.9)	0.11 (0.10-0.13)	
3-5	783/5761 (13.6)	292/13149 (2.2)	0.11 (0.10-0.13)	
NIHSS score at 24h				0.1984
0-4	15/2768 (0.5)	9/5025 (0.2)	0.25 (0.10-0.64)	
5-9	46/1333 (3.5)	43/6326 (0.7)	0.15 (0.10-0.23)	
10-20	315/2935 (10.7)	206/11901 (1.7)	0.11 (0.09-0.13)	
21-	743/3222 (23.1)	245/3592 (6.8)	0.16 (0.14-0.18)	
Statin				0.7315
No	692/3626 (19.1)	128/2429 (5.3)	0.15 (0.12-0.18)	
Yes	883/9378 (9.4)	447/26898 (1.7)	0.13 (0.11-0.14)	
Covid19 pandemic				0.9504
Before	1535/12894 (11.9)	568/28270 (2.0)	0.11 (0.10-0.13)	
During	172/1219 (14.1)	66/2828 (2.3)	0.11 (0.08-0.15)	

Abbreviations: BT, bridging thrombolysis; CE, cardioembolism; DET, direct endovascular therapy; EVT, endovascular therapy; ICH, intracranial hemorrhage; IRT, inpatient rehabilitation therapy; LAA, large artery atherosclerosis; mRS, modified ranking score; NIHSS, National Institutes of Health Stroke Scale; SAO, small artery occlusion; SOC, stroke of other determined cause; SUC, stroke of undetermined cause.

TableS9. Clinical characteristics of patients with initial NIHSS score ≥ 21

	Thrombolysis-only group			EVT group	
	No. of patients, (%)	No. of death,(%)		No. of patients, (%)	No. of death,(%)
Age group					
< 65y/75y	1979 (21.4)	112 (5.7)		7116 (70.5)	590 (8.3)
≥ 65y/75y	7252 (78.6)	604 (8.3)		2972 (29.5)	336 (11.6)
ICH					
No	8467 (91.7)	608 (7.2)		8986 (89.1)	732 (8.2)
Yes	764 (8.3)	108 (14.1)		1100 (10.9)	189 (17.2)

Note: The number and percentage of age≥65 in thrombolysis-only group were 105069 (55.7%);the number and percentage of age≥75 in EVT group were 10514 (23.3%).

TableS10. Sensitivity analyses of Cox regression models in patients with only thrombolysis

a. Cox regression model in patients with follow-up time ≥ 1 days

		Unadjusted			Adjusted [aa]	
	Event, n(%)	HR (95% CI)	p Value		HR (95% CI)	p Value
IRT-	1270 (1.7)	1 (Reference)	NA		1 (Reference)	NA
IRT+	599 (0.6)	0.26 (0.23-0.29)	<0.0001		0.19 (0.17-0.21)	<0.0001

[aa] The model adjusted for patient age, hospital level, TOAST, reperfusion time, use of antiplatelet within 48h, use of statin, NIHSS score at 24h, NIHSS score change at 24h, and intracranial hemorrhage, shown in eTable5 in the supplement. 159929 patients were included in multi-variable model.

b. Cox regression model in patients with follow-up time ≤ 6 days

		Unadjusted			Adjusted [bb]	
	Event, n(%)	HR (95% CI)	p Value		HR (95% CI)	p Value
IRT-	1517 (4.1)	1 (Reference)	NA		1 (Reference)	NA
IRT+	355 (1.4)	0.28 (0.25-0.31)	<0.0001		0.36 (0.31-0.41)	<0.0001

[bb] The model adjusted for patient age, hospital level, TOAST, reperfusion time, use of antiplatelet within 48h, use of statin, NIHSS score at 24h, NIHSS score change at 24h, and intracranial hemorrhage, shown in eTable5 in the supplement. 51135 patients were included in multi-variable model.

c. Time-dependent Cox regression model

		Unadjusted			Adjusted [cc]	
	Event, n(%)	HR (95% CI)	p Value		HR (95% CI)	p Value
IRT-	1908 (2.3)	1 (Reference)	NA		1 (Reference)	NA
IRT+	667 (0.6)	0.20 (0.19-0.22)	<0.0001		0.19 (0.17-0.22)	<0.0001

[cc] The time-dependent Cox regression model adjusted for patient age, hospital level, TOAST, reperfusion time, use of antiplatelet within 48h, use of statin, NIHSS score at 24h, NIHSS score change at 24h, and intracranial hemorrhage, shown in eTable5 in the supplement and sex, region, covid19 pandemic, and thrombolysis drug. 141012 patients were included in multi-variable model.

TableS11. Sensitivity analyses of Cox regression models in patients with EVT

a. Cox regression model in patients with follow-up time ≥ 1 days

		Unadjusted			Adjusted [aa]	
	Event, n(%)	HR (95% CI)	p Value		HR (95% CI)	p Value
IRT-	1298 (10.8)	1 (Reference)	NA		1 (Reference)	NA
IRT+	561 (1.9)	0.13 (0.11-0.14)	<0.0001		0.13 (0.12-0.15)	<0.0001

[aa] The model adjusted for patient age, hospital level, TOAST, reperfusion time, reperfusion status, use of antiplatelet within 48h, use of statin, NIHSS score at 24h, NIHSS score change at 24h, and intracranial hemorrhage, shown in eTable6 in the supplement. 31956 patients were included in multi-variable model.

b. Cox regression model in patients with follow-up time ≤ 6 days

		Unadjusted			Adjusted [bb]	
	Event, n(%)	HR (95% CI)	p Value		HR (95% CI)	p Value
IRT-	1342 (19.1)	1 (Reference)	NA		1 (Reference)	NA
IRT+	312 (5.3)	0.19 (0.17-0.21)	<0.0001		0.27 (0.23-0.32)	<0.0001

[bb] The model adjusted for patient age, hospital level, TOAST, reperfusion time, reperfusion status, use of antiplatelet within 48h, use of statin, NIHSS score at 24h, NIHSS score change at 24h, and intracranial hemorrhage, shown in eTable6 in the supplement. 8855 patients were included in multi-variable model.

c. Time-dependent Cox regression model

		Unadjusted			Adjusted [cc]	
	Event, n(%)	HR (95% CI)	p Value		HR (95% CI)	p Value
IRT-	1707 (12.1)	1 (Reference)	NA		1 (Reference)	NA
IRT+	634 (2.0)	0.11 (0.10-0.13)	<0.0001		0.13 (0.12-0.15)	<0.0001

[cc] The model adjusted for patient age, hospital level, TOAST, reperfusion time, reperfusion status, use of antiplatelet within 48h, use of statin, NIHSS score at 24h, NIHSS score change at 24h, and intracranial hemorrhage, shown in eTable6 in the supplement, and sex, region, covid19 pandemic and EVT intervention type (bridging thrombolysis vs. direct endovascular therapy). 30268 patients were included in multi-variable model.

TableS12. Confounders of Cox regression model in thrombolysis-only group

Items	Details
Age group	(1) 14-60 years; (2) 61-80 years; (3) 71-99 years
Hospital level	(1) Class-A hospitals; (2) Class-B hospitals; (3) Class-C hospitals
TOAST	(1) Large artery atherosclerosis, or cardioembolism;(2) small artery occlusion, or stroke of other determined cause, or stroke of undetermined cause;
Reperfusion time	(1) ONT ≤ 149 mins; (2) ONT ≥ 150mins
Antiplatelet within 48h	(1) No, or Unknown; (2) Yes
Statin	(1) No; (2) Yes
NIHSS score at 24h	(1) 0-7; (2)≥8
NIHSS score change at 24h	(1) Severe deterioration; (2) Non-severe deterioration
Intracranial hemorrhage	(1) No; (2) Yes

TableS13. Confounders of Cox regression model in EVT group

Items	Details
Age group	(1) 14-60 years; (2) 61-80 years; (3) 81-99 years
Hospital level	(1) Class-A hospitals; (2) Class-B hospitals, or Class-C hospitals
TOAST	(1) Large artery atherosclerosis, or cardioembolism;(2) Small artery occlusion, or stroke of other determined cause, or stroke of undetermined cause;
Reperfusion time	(1) OPT ≤ 269 mins; (2) OPT ≥ 270mins
Reperfusion status	(1) modified TICI: 0,1, or 2a; (2) modified TICI: 2b or 3; (3)Unknown
Antiplatelet within 48h	(1) No, or Unknown; (2) Yes
Statin	(1) No; (2) Yes
NIHSS score at 24h	(1) 0-7; (2)≥8
NIHSS score change at 24h	(1) Severe deterioration; (2) Non-severe deterioration
Intracranial hemorrhage	(1) No; (2) Yes

FigureS1. Data source of our study

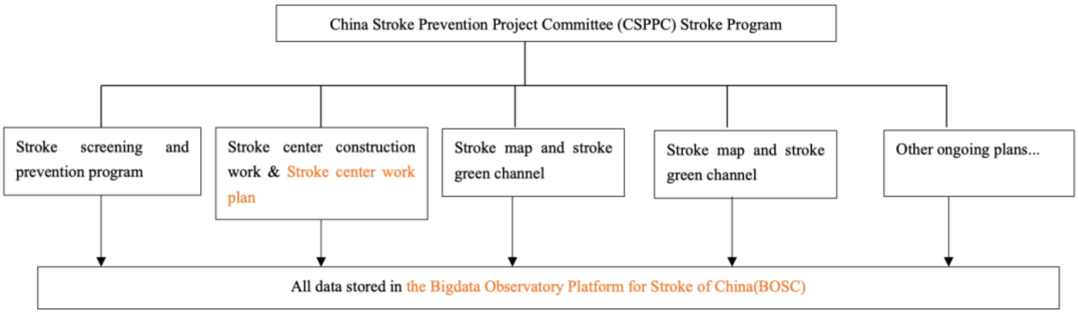

Note: All data of the CSPPC Stroke Program are collected and stored in the Bigdata Observatory Platform for Stroke of China (BOSC, https://www.chinasdc.cn).

FigureS2. The distribution of propensity scores in unmatched and matched patients in thrombolysis-only group

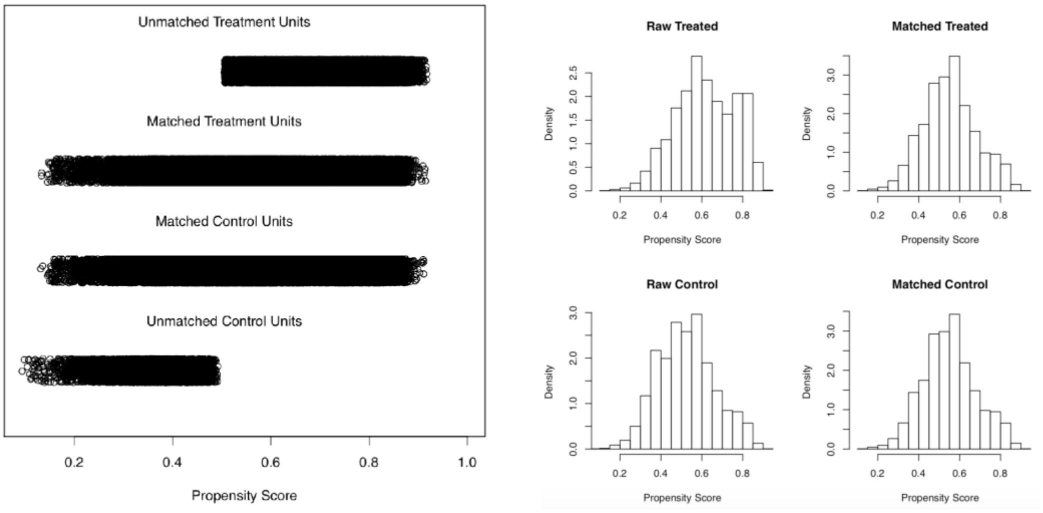

FigureS3. The distribution of propensity scores in unmatched and matched patients in EVT group

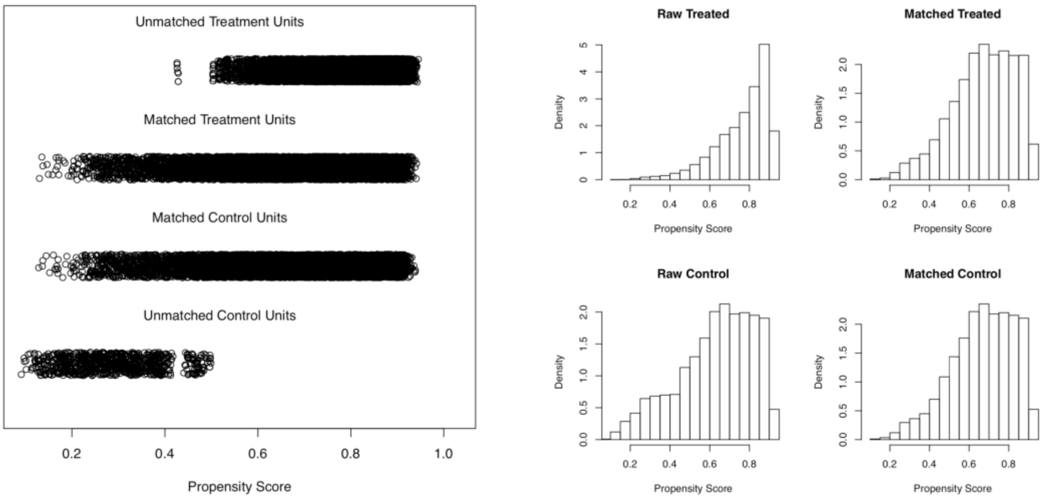

Supplement: Supplementary file 1 [file Data_Sheet_1.pdf]
